# The Low-Molecular Weight Protein Arginine Phosphatase PtpB Affects Nuclease Production, Cell Wall Integrity, and Uptake Rates of *Staphylococcus aureus* by Polymorphonuclear Leukocytes

**DOI:** 10.3390/ijms22105342

**Published:** 2021-05-19

**Authors:** Mohamed Ibrahem Elhawy, Virginie Molle, Sören L. Becker, Markus Bischoff

**Affiliations:** 1 Institute of Medical Microbiology and Hygiene, Saarland University, 66421 Homburg, Germany; Mohamed.Elhawy@uks.eu (M.I.E.); soeren.becker@uks.eu (S.L.B.); 2 Department of Pathology, Faculty of Veterinary Medicine, Zagazig University, Zagazig 44511, Egypt; 3 Laboratory of Pathogen Host Interactions, Université de Montpellier, CNRS, UMR 5235, 34095 Montpellier, France; virginie.molle@umontpellier.fr

**Keywords:** *Staphylococcus aureus*, low-molecular-weight protein arginine phosphatase, PtpB, nuclease, whole blood phagocytosis assay, cell wall integrity, gene transcription, Triton X-100 induced autolysis, innate immunity

## Abstract

The epidemiological success of *Staphylococcus aureus* as a versatile pathogen in mammals is largely attributed to its virulence factor repertoire and the sophisticated regulatory network controlling this virulon. Here we demonstrate that the low-molecular-weight protein arginine phosphatase PtpB contributes to this regulatory network by affecting the growth phase-dependent transcription of the virulence factor encoding genes/operons *aur*, *nuc*, and *psmα*, and that of the small regulatory RNA *RNAIII*. Inactivation of *ptpB* in *S. aureus* SA564 also significantly decreased the capacity of the mutant to degrade extracellular DNA, to hydrolyze proteins in the extracellular milieu, and to withstand Triton X-100 induced autolysis. SA564 Δ*ptpB* mutant cells were additionally ingested faster by polymorphonuclear leukocytes in a whole blood phagocytosis assay, suggesting that PtpB contributes by several ways positively to the ability of *S. aureus* to evade host innate immunity.

## 1. Introduction

*Staphylococcus aureus* is a major bacterial pathogen in humans and animals [1]. The ability of this Gram-positive bacterium to respond accurately to changing environments is one of the prerequisites for its success as a versatile pathogen in mammals [2]. The opportunistic pathogen is equipped with an armamentarium of virulence factors and a sophisticated network of regulatory molecules allowing it to control/fine-tune the expression of its virulon in a way to rapidly respond to changing conditions [3]. While virulence factor synthesis in *S. aureus* is mainly driven by one component systems such as the Sar family of DNA binding proteins and two-component system (TCS) response regulators [4], phosphatases are also known to contribute to this network. Earlier work demonstrated, for instance, that the serine/threonine phosphatase Stp1 directly contributes to virulence factor synthesis and infectivity of *S. aureus* by promoting the transcription of the α-hemolysin encoding gene *hla* [5]. More recent data indicate that the low-molecular-weight protein arginine phosphatase (LMW-PAP) PtpB might also contribute to staphylococcal pathophysiology by modulating the arginine phosphorylation states of regulators such as the stress response regulator CtsR and the global regulator MgrA, particularly in response to oxidative stress [6,7]. A deletion of *ptpB* in the *S. aureus* clinical isolate SA564 was additionally shown lately to reduce the capacity of the mutant to survive inside of macrophages and to cause infection in a murine-based *S. aureus* abscess model [8]. PtpB might thereby form a regulatory circuit with McsB, the presumed protein arginine kinase of *S. aureus*, which is part of the *clpC* operon and known to affect the hemolytic and proteolytic activity of this bacterium [9]. The McsB homolog in *Bacillus subtilis* was shown to modulate the activity of McsB-targeted regulators by phosphorylating arginine residues within the DNA-binding domains and by marking proteins and aberrant polypeptide molecules for degradation [10,11]; similar functions are assumed for McsB in *S. aureus* [6]. Thus, by counteracting McsB-dependent arginine phosphorylation of the aforementioned global regulators, PtpB might affect the activity and stability of these transcription factors and thus the expression of stress response genes and virulence factors that are directly and/or indirectly controlled by these regulatory molecules. Additionally, PtpB might interfere with the degradation process of proteins phosphorylated at arginine residues. Notably, a functional classification of arginine phosphorylated proteins found in in vitro cultured *S. aureus* cells carried out by Junker et al. [6] revealed that nearly half (47%) of the identified arginine phosphoproteins in the *ptpB* mutant could be associated with virulence functions, supporting the idea that PtpB contributes to the regulatory network controlling the virulence factor synthesis in this bacterial pathogen.

In this study, we further strengthen this assumption by demonstrating that inactivation of *ptpB* in *S. aureus* strain SA564—a whole genome sequenced clinical human isolate of the worldwide distributed clonal complex 5 that harbors functional *agr* and *sigB* operons and a *saeS*^L^ allele [12,13]—affects the transcription of the virulence factor encoding genes *aur* (encoding the zinc-metalloprotease aureolysin), *nuc* (encoding thermonuclease) and *psmα* (encoding the phenol-soluble modulins α1-4), and of the small regulatory RNA *RNAIII*, one of the master regulators controlling exoprotein synthesis in this bacterium [14], which also encodes a protein, δ-hemolysin (Hld). Deletion of *ptpB* also impedes the capacity of *S. aureus* to evade phagocytosis by polymorphonuclear leukocytes (PMNs), to withstand Triton X-100 induced autolysis or lysostaphin mediated lysis, and decreases the exonuclease- and exoprotease activities of this bacterium.

## 2. Results and Discussion

### 2.1. PtpB Affects the Ability of S. aureus to Evade Phagocytosis by PMNs

**Figure 1:**
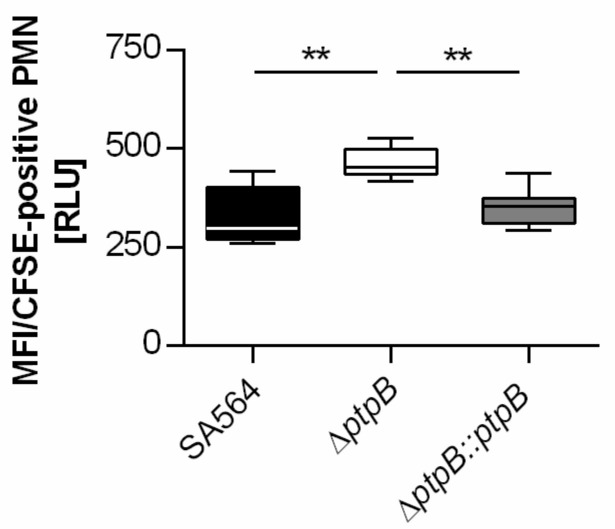
Effect of PtpB on phagocytosis of *S. aureus* SA564 by polymorphonuclear leukocytes (PMNs). Carboxy fluorescein diacetate succinimidyl ester (CFSE)-stained cells of *S. aureus* SA564 (black boxes), SA564 Δ*ptpB* (white boxes), and SA564 Δ*ptpB::ptpB* (gray boxes) were added to lithium heparin anticoagulated fresh human blood at a multiplicity of infection of 50 per PMN, and incubated for 30 min at 37 °C and 1000 rpm. Attachment/uptake of bacteria by PMNs was analyzed by flow cytometry as outlined in Material and Methods. The data are representative of three biological replicates carried out in triplicate. Data are presented as box and whisker plots showing the interquartile range (25–75%; box), median (horizontal line), and whiskers (bars; min/max). ** *p* < 0.01 (Kruskal–Wallis test followed by Dunn’s post hoc test).

Recent work demonstrated that PtpB contributes to the ability of *S. aureus* to survive inside macrophages [8]. However, it remains unknown whether PtpB also supports the capacity of the pathogen to evade innate immunity. In order to address this issue, we first investigated the impact of a *ptpB* deletion on attachment/phagocytosis of *S. aureus* SA564 by PMNs in whole blood (Figure 1).

When human blood was supplemented with fluorescent-labeled bacteria at a multiplicity of infection (MOI) of 50 per PMNs, a clear difference in uptake rates of *ptpB* negative and positive *S. aureus* SA564 cells by human PMNs was observed (Figure 1). After 30 min of coincubation in human whole blood, CFSE-stained SA564 isolates harboring a functional *ptpB* (wild-type and *cis*-complemented derivative) demonstrated significantly decreased rates of attachment/phagocytosis by PMNs compared to the CFSE-stained SA564 derivative lacking *ptpB* (SA564 Δ*ptpB*). These findings suggest that PtpB mediates a relevant protective effect against phagocytosis by PMNs in blood.

### 2.2. PtpB Promotes the Transcription and Secretion of Nuclease in S. aureus

**Figure 2:**
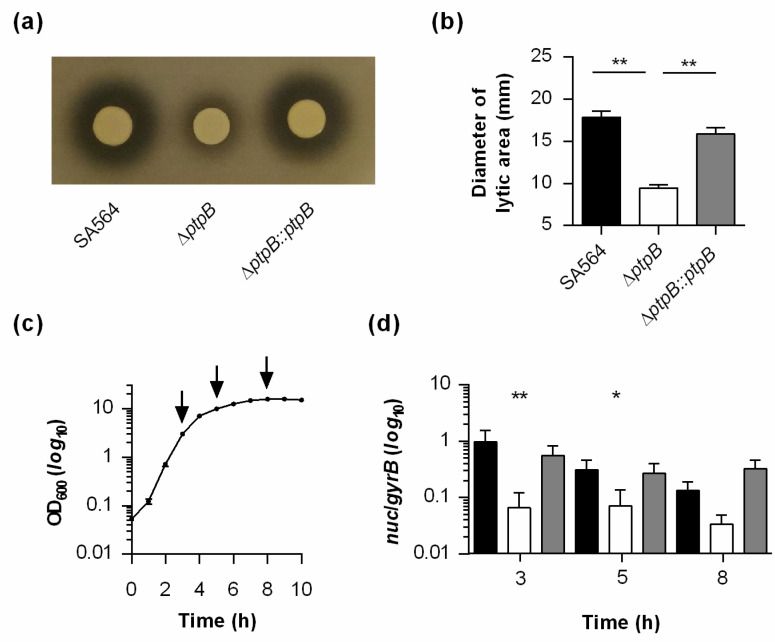
Effect of PtpB on extracellular DNase activity and *nuc* transcription of *S. aureus* SA564. (**a**,**b**) Impact of PtpB on extracellular DNase activity. Overnight cultures of *S. aureus* strain SA564 (black bars), its isogenic Δ*ptpB* mutant (white bars), and the *cis*-complemented derivative (gray bars) were normalized with fresh tryptic soy broth (TSB) medium to an optical density at 600 nm (OD_600_) of 12, and 5 µL of the suspensions spotted on DNase-Test-Agar plates. Inoculated plates were cultured for 24 h at 37 °C, and lytic areas were determined. (**a**) Representative image of one experiment. (**b**) Diameter of lytic areas. The data presented are the mean + SD of three biological experiments done in duplicate. ** *p* < 0.01 (Kruskal–Wallis test followed by Dunn’s post hoc test). (**c**) Growth kinetics of *S. aureus* strain SA564 in TSB. Cells were cultured at 37 °C and 225 rpm at a culture to flask volume of 1:10. Data represent the mean OD_600_ readings ± SD at the time points indicated (*n* = 3). Time points of sampling for the transcriptional analyses are indicated by arrows. (**d**) Impact of PtpB on transcription of the thermonuclease encoding *nuc* gene. Quantitative transcript analyses of *nuc* by qRT-PCR in SA564 (black bars), SA564 Δ*ptpB* (white bars), and SA564 Δ*ptpB::ptpB* (gray bars) cells grown to the time points indicated. Transcript rates were quantified in reference to the transcription of gyrase B (in copies per copy of *gyrB*). Data are presented as mean + SD of five biological replicates. * *p* < 0.05; ** *p* < 0.01 (Kruskal–Wallis test followed by Dunn’s post hoc test between wild-type and mutants at a given time point).

Neutrophils are the main pathogen-fighting immune cells in our blood system [15]. Besides their capacity to ingest circulating pathogens, activated neutrophils exert a number of cytotoxic functions such as the production of reactive oxygen and nitrogen species, the release of antimicrobial peptides, and the formation of extracellular DNA nets called neutrophil extracellular traps (NETs) [15]. The latter bacteria-capturing and killing mechanism is counteracted by *S. aureus,* among others, via the secretion of nucleases and the extracellular adherence protein (Eap), which degrade and aggregate NETs, respectively [16,17]. We have recently shown that PtpB supports the capacity of *S. aureus* to cope with oxidative and nitrosative stress [8]. To test whether PtpB might also interfere with other neutrophil-derived cytotoxic activities, we next tested whether the LMW-PAP might modulate the capacity of *S. aureus* to degrade extracellular DNA (Figure 2).

When cell suspensions of SA564 and the *cis*-complemented Δ*ptpB::ptpB* derivative were spotted on DNase test agar plates and incubated at 37 °C for 24 h, clearly visible lytic areas around the bacterial growth areas were observed that were in comparable ranges (Figure 2a,b). However, when equal volumes of SA564 Δ*ptpB* cell suspensions were spotted onto the DNase test agar plates, significantly smaller lytic areas surrounding the bacterial growth zones were observed, suggesting that the Δ*ptpB* mutant produces and/or secretes lower amounts of nucleases into the extracellular milieu.

To elucidate whether the reduced nuclease activity observed with the Δ*ptpB* mutant might be due to PtpB-induced changes in *nuc* transcription, we next assayed the transcription rates of *nuc* in SA564 and its Δ*ptpB* derivative upon growth in tryptic soy broth (TSB) over time. Specifically, total RNAs were obtained from cell populations grown for 3 h (exponential growth phase), 5 h (transition phase), and 8 h (early stationary phase), respectively (Figure 2c). In line with our DNase activity findings, we observed a significantly reduced transcription of *nuc* in SA564 Δ*ptpB* cells at two of the three growth stages analyzed, if compared to the wild-type transcript level, while cells of the *cis*-complemented derivative produced *nuc* transcript level that were comparable to the wild-type. Taken together, these findings suggest that PtpB contributes positively to the exonuclease activity of *S. aureus* by supporting *nuc* transcription.

### 2.3. PtpB Promotes the Transcription of the Aureolysin Encoding Gene aur in S. aureus

**Figure 3:**
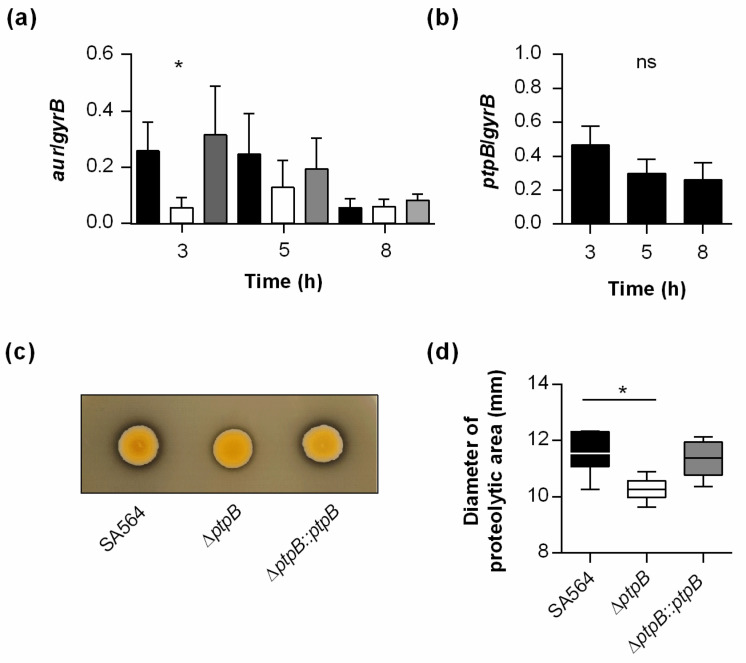
Effect of a *ptpB* deletion on the transcription of *aur* and the proteolytic activity of *S. aureus* SA564. (**a**) Quantitative transcript analyses of *aur* by qRT-PCR in SA564 (black bars), SA564 Δ*ptpB* (white bars), and SA564 Δ*ptpB::ptpB* (gray bars) cells grown in tryptic soy broth (TSB) at 37 °C and 225 rpm to the time points indicated. Transcript rates were quantified in reference to the transcription of gyrase B (in copies per copy of *gyrB*). Data are presented as mean + SD of four to five biological replicates. * *p* < 0.05 (Kruskal–Wallis test followed by Dunn’s post hoc test between wild-type and mutants at a given time point). (**b**) Quantitative transcript analyses of *ptpB* by qRT-PCR in SA564 cells grown in TSB. Sampling and transcription rate quantifications were done as described above. Data are presented as mean + SD of five biological replicates. (**c**,**d**) Impact of PtpB on extracellular protease activity. Overnight cultures of *S. aureus* strain SA564 (black bars), its isogenic Δ*ptpB* mutant (white bars), and the *cis*-complemented SA564 Δ*ptpB::ptpB* derivative (gray bars) were normalized with fresh TSB medium to an OD_600_ of 12, and 2 µL of the suspensions spotted on TSA plates supplemented with 10% skim milk. Inoculated plates were cultured for 48 h at 37 °C, and proteolytic areas were determined. (**c)** Representative image of one experiment. (**d**) Diameter of proteolytic areas. The data presented are the mean + SD of three biological experiments done in duplicate. ns, not significant; * *p* < 0.05 (Kruskal–Wallis test followed by Dunn’s post hoc test).

Another major virulence determinant interfering with innate host immunity and supporting the survival of *S. aureus* in blood is the zinc-metalloprotease aureolysin, which cleaves, among others, various factors of the human complement system [18]. To test whether PtpB might aid the immune evasion of *S. aureus* by modulating aureolysin expression, we tested the transcription of *aur* during growth in TSB (Figure 3a). This transcriptional analysis revealed a significantly decreased number of *aur* transcripts in exponential growth phase cells of the *ptpB* deletion mutant when compared with cells of the wild-type and the *cis*-complemented derivative harvested at the same growth stage, respectively. However, *aur* transcript rates were rather comparable between wild-type, Δ*ptpB* mutant, and *cis*-complemented cells obtained from later growth stages (i.e., 5 and 8 h of growth), suggesting that PtpB contributes to *aur* expression primarily in *S. aureus* exponential growth phase cells.

To get an idea about whether the impact of PtpB on *aur* transcription might correlate with the expression of the LMW-PAP, we determined the transcription rates of *ptpB* in SA564 during growth in TSB (Figure 3b). These analyses revealed that *ptpB* is transcribed on a rather constant level throughout growth (fold changes in relative transcription rates between growth stages < 2), suggesting that the growth phase-dependent effect of PtpB on *aur* transcription is not likely to be determined by the expression rates of the LMW-PAP during growth.

Besides its complement factors degrading functions [18], aureolysin is also known for its capacity to process and thus activate serine protease SspA (syn. V8 protease), another major exoprotease produced by *S. aureus*, which in turn cleaves the propeptide from the SspB zymogen to create the active cysteine protease SspB (syn. staphopain), a process also known as staphylococcal proteolytic cascade [19].

Wondering whether PtpB might also affect the exoproteolytic capacity of *S. aureus*, we conducted a series of skim milk agar-based protease assays (Figure 3c,d). Stationary phase cell suspensions of SA564 normalized to an OD_600_ of 12 spotted onto the skim milk supplemented tryptic soy agar (TSA) plates produced only very small proteolytic areas surrounding the bacterial growth zones after 48 h of incubation at 37 °C (Figure 3c). However, when normalized stationary phase cell suspensions of SA564 Δ*ptpB* were spotted onto the skim milk TSA plates, significantly smaller proteolytic areas were observed, while cell suspensions of the *cis*-complemented SA564 Δ*ptpB::ptpB* produced proteolytic areas on skimmed milk TSA plates that were in a comparable range to those seen with the wild-type cultures (Figure 3c,d). These findings suggest that PtpB affects the proteolytic capacity of *S. aureus*, potentially via the modulation of *aur* transcription.

### 2.4. PtpB Reduces the Autolytic Activity of S. aureus

**Figure 4:**
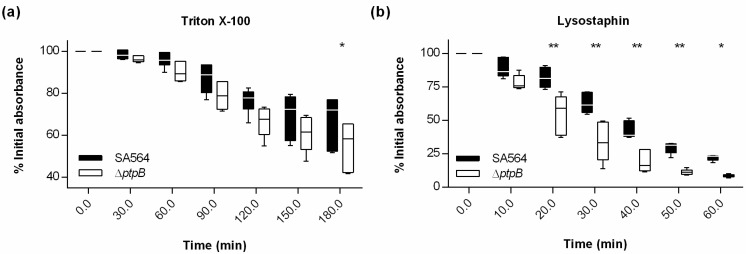
Effect of a *ptpB* deletion on the Triton X-100 induced autolysis and lysostaphin-mediated lysis of *S. aureus* SA564. (**a**) Cells of *S. aureus* strain SA564 (black symbols) and its isogenic Δ*ptpB* mutant (white symbols) were cultured in TSB at 37 °C and 225 rpm to an OD_600_ of 0.7, washed twice in ice-cold water, and resuspended in 0.05 M Tris-HCl (pH 7.2) containing 0.01% (vol/vol) Triton X-100. Triton X-100 induced autolysis was measured as the decline of optical density at 600 nm (OD_600_) versus time and is expressed as the percent of the initial optical density (*n* = 6 biological replicates). (**b**) Effect of a *ptpB* deletion on the lysostaphin-mediated lysis. Cells of *S. aureus* strain SA564 (black symbols) and its isogenic Δ*ptpB* mutant (white symbols) were cultured in TSB at 37 °C and 225 rpm to an OD_600_ of 3, washed twice in PBS, and resuspended in PBS to an OD_600_ of 1. The PBS cell suspensions were supplemented with 250 ng/mL lysostaphin and incubated under static conditions at 30 °C for 60 min. Lysostaphin-mediated lysis was measured as the decline of OD_600_ versus time and is expressed as the percent of the initial optical density (*n* = 5 biological replicates). Data are presented as box and whisker plots showing the interquartile range (25–75%; box), median (horizontal line), and whiskers (bars; min/max). * *p* < 0.05; ** *p* < 0.01 (Ordinary two-way ANOVA followed by Holm–Sidak’s multiple comparison test).

Activated neutrophils secrete, among others, antimicrobial peptides (e.g., defensins) which exert potent in vitro microbicidal activity against *S. aureus* through mechanisms involving cell membrane destabilization, interference with intracellular targets, and activation of autolysins [20]. To test whether and how PtpB might influence the autolytic behavior of *S. aureus*, we next studied the impact of the *ptpB* deletion on the autolytic behavior of SA564 in Triton X-100- and lysostaphin-induced lysis assays, respectively (Figure 4).

When washed exponential growth phase cells of SA564 and its Δ*ptpB* mutant were challenged with a low dose of Triton X-100, respectively, cells of the *ptpB* deletion mutant autolyzed to a significantly larger extent after 3 h of coincubation with the detergent than wild-type cells (Figure 4a). A similar behavior was observed when SA564 and its Δ*ptpB* mutant were challenged with the glycyl-glycyl endopeptidase lysostaphin, a *S. aureus* pentaglycine cross-bridges cleaving exoenzyme from *Staphylococcus simulans* bv. *staphylolyticus* [21]. Washed and PBS resuspended late exponential growth phase cells of the *ptpB* deletion mutant lysed again significantly faster than the wild-type cells (Figure 4b), indicating that PtpB is likely to affect the cell wall composition of *S. aureus*. One possible explanation for both observations is that PtpB might interfere with the production and/or activation of autolysins (e.g., endogenous murein hydrolases), as has been suggested for the lysostaphin resistance of some glycopeptide-intermediate-resistant *S. aureus* (GISA) [22].

**Figure 5:**
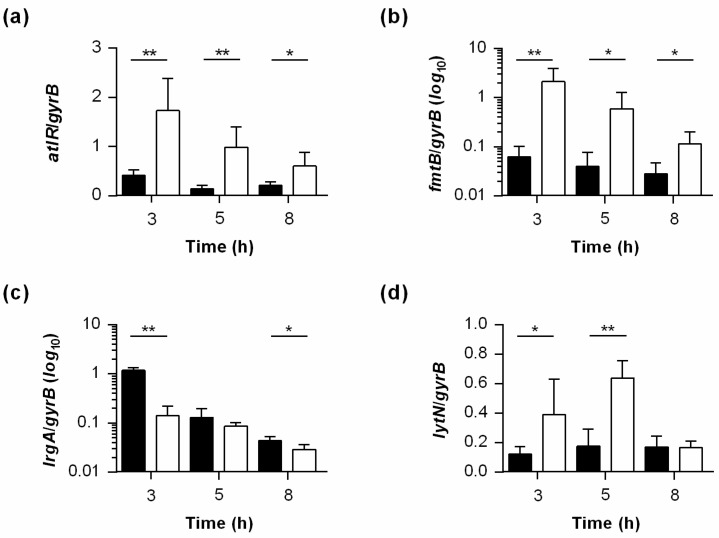
Effect of a *ptpB* deletion on the transcription of MgrA-regulated genes affecting autolysis of *S. aureus*. Quantitative transcript analyses of MgrA regulated genes by qRT-PCR in SA564 (black bars) and SA564 Δ*ptpB* (white bars) cells grown in TSB at 37 °C and 225 rpm to the time points indicated. Growth-phase-dependent transcript rates of MgrA-regulated genes *atlR* (**a**), *ftmB* (**b**), *lrgA* (**c**), and *lytN* (**d**). Transcript rates were quantified in reference to the transcription of gyrase B (in copies per copy of *gyrB*). Data are presented as mean + SD of five biological replicates. * *p* < 0.05; ** *p* < 0.01 (Mann–Whitney-U test between wild-type and mutant at a given time point).

As previous studies have already demonstrated that the global regulator MgrA acts as a repressor of autolysins in *S. aureus* [23,24], and that MgrA serves as a substrate for PtpB [6,7], it was tempting to speculate that PtpB might affect the autolytic behavior of *S. aureus* via modulation of MgrA activity. In order to address this hypothesis, we first studied the impact of the *ptpB* deletion on the transcription of MgrA regulated genes [25,26], focusing on genes whose products are involved in the autolytic behavior of *S. aureus* (Figure 5). Specifically, the transcription of *atlR* (encoding a MarR family transcriptional regulator repressing *atl* transcription), *fmtB* (encoding a cell wall-anchored protein involved in methicillin resistance and cell wall biosynthesis), *lrgA* (encoding the holin-like murein hydrolase regulator LrgA), and *lytN* (encoding the cell-wall hydrolase LytN) was analyzed, all of which were reported to directly or indirectly affect the autolytic behavior of *S. aureus* [27,28,29].

Notably, all four MgrA-regulated genes were transcribed in a significantly different manner in SA564 Δ*ptpB* cells than in wild-type cells. Importantly, genes reported to be repressed by MgrA (i.e., *atlR*, *fmtB*, and *lytN*) were all transcribed on higher levels in the *ptpB* deletion mutant, while transcription of *lrgA*, which is positively affected by MgrA activity [25], was increased in wild-type cells throughout growth. However, while transcription of *atlR*, *fmtB*, and *lrgA* was affected by the *ptpB* deletion basically at all three growth stages (Figure 5a–c), this was not the case with *lytN*. Transcription of the cell-wall hydrolase encoding gene was only affected by the *ptpB* deletion during exponential growth phase and transition phase, but not in the early stationary phase (Figure 5d), suggesting that *lytN* expression in the stationary phase cells is dominated by PtpB/MgrA-independent mechanisms. Taken together, these data indicate that the PtpB-driven dephosphorylation of MgrA phosphoarginine residues observed by Junker et al. [6] is likely to affect MgrA activity, and supports our hypothesis that PtpB interferes with autolysis of *S. aureus* via modulation of MgrA activity.

### 2.5. PtpB Does Not Alter MgrA Activity per Se

**Figure 6:**
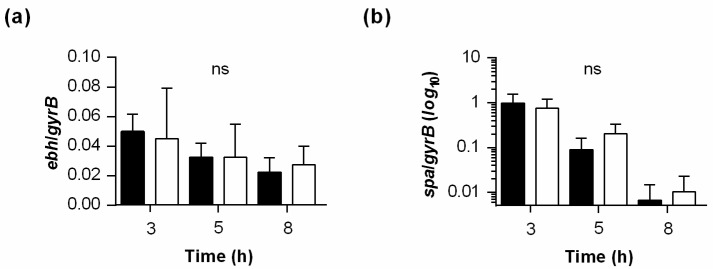
Effect of a *ptpB* deletion on the transcription of MgrA-repressed surface factors encoding genes. Quantitative transcript analyses of MgrA-regulated genes *ebh* (**a**) and *spa* (**b**) by qRT-PCR in SA564 (black bars) and SA564 Δ*ptpB* (white bars) cells grown in TSB at 37 °C and 225 rpm to the time points indicated. Transcript rates were quantified in reference to the transcription of gyrase B (in copies per copy of *gyrB*). Data are presented as mean + SD of five biological replicates. ns, not significant (Mann–Whitney-U test between wild-type and mutant at a given time point).

Given that MgrA also promotes the transcription of *aur* and *nuc* [25], which were also found to be transcribed in significantly higher levels in wild-type cells when compared to Δ*ptpB* mutant cells (Figure 2c and Figure 3), we wondered whether PtpB might affect the transcription of the whole MgrA regulon. In order to address this hypothesis, we additionally tested the transcription of two MgrA-regulated surface factor encoding genes, *ebh* (encoding extracellular matrix-binding protein Ebh) and *spa* (encoding immunoglobulin G binding protein A), both of which are known to be strongly repressed by MgrA activity on the transcriptional level [26]. In contrast to findings obtained for the MgrA-regulated genes affecting autolysis (Figure 5), we surprisingly observed no clear differences in transcription rates of *ebh* and *spa* between the wild-type and the *ptpB* deletion mutant at any time point analyzed (Figure 6).

As MgrA directly represses *ebh* transcription [26], our findings presented here indicate that PtpB-driven dephosphorylation of MgrA does not alter the activity of the transcription factor towards its regulon per se, but might be important for the expression of a subset of MgrA-regulated factors, particularly of those involved in autolysis. One explanation for our observations might be that PtpB-mediated dephosphorylation of MgrA arginine residues influences the activity of the regulator by differentiating the binding specificity among target gene promoters, depending on the phosphorylation status at arginine residues. In such a scenario, target genes with a high binding affinity for MgrA would not be affected in their expression by the arginine phosphorylation status of the regulator and thus would not display a change in transcription depending on the PtpB status of the cell.

### 2.6. PtpB Alters the Transcription of Some but Not All SarA Regulated Genes

**Figure 7:**
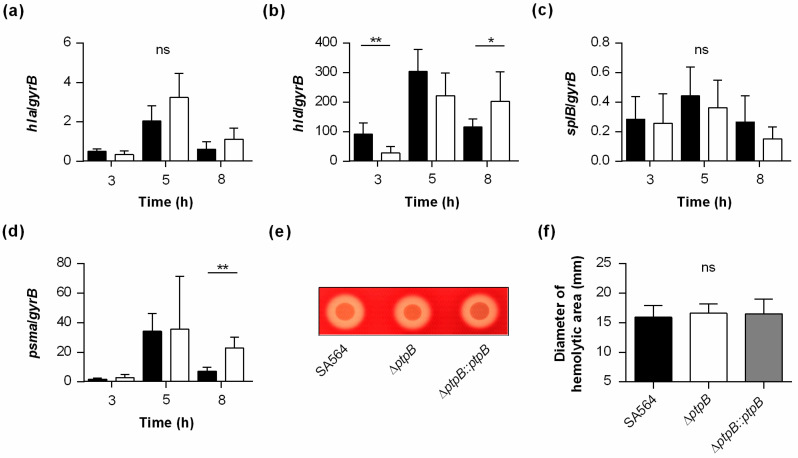
Effect of a *ptpB* deletion on the transcription of SarA- and/or AgrA-regulated genes in *S. aureus* SA564. (**a**–**d**) Quantitative transcript analyses of SarA- and/or AgrA-regulated genes by qRT-PCR in SA564 (black bars) and SA564 Δ*ptpB* (white bars) cells grown in TSB at 37 °C and 225 rpm to the time points indicated. Growth phase-dependent transcript rates of Sar box containing genes *hla* (**a**), *hld* (**b**), and *splB* (**c**), and of the AgrA-driven *psma* cluster (**d**). Transcript rates were quantified in reference to the transcription of gyrase B (in copies per copy of *gyrB*). Data are presented as mean + SD of five biological replicates. * *p* < 0.05; ** *p* < 0.01; ns, not significant (Mann–Whitney-U test between wild-type and mutant at a given time point). (**e**,**f**) Overnight cultures of *S. aureus* strain SA564 (black bar), its isogenic Δ*ptpB* mutant (white bar), and the *cis*-complemented SA564 Δ*ptpB::ptpB* derivative (gray bar) were normalized with fresh TSB medium to an OD_600_ of 12, and 5 µL of the suspensions spotted on sheep blood agar plates. Inoculated plates were cultured for 24 h at 37 °C, and hemolytic areas were determined. (**e**) Representative image of one experiment. (**f**) Diameter of hemolytic areas. The data presented are the mean + SD of three biological experiments done in duplicate. ns, not significant (Kruskal–Wallis test followed by Dunn’s post hoc test).

Transcription of *aur*, *lrgA*, and *nuc* is also reported to be affected by SarA [30,31,32], suggesting that PtpB might also interfere with the transcription of the aforementioned genes via modulation of SarA activity, although SarA was not identified as a direct substrate for McsB/PtpB [6,7]. To test whether and how PtpB affects the transcription of the SarA regulon in SA564, we assayed the transcription rates of three additional SarA target genes, *hla* (encoding α-hemolysin), *hld* (encoding δ-hemolysin), and *splB* (encoding serine protease SplB; Figure 7) [32,33,34], which all contain a putative Sar box within the gene/operon’s promoter region [32]. Similar to the situation seen with MgrA-regulated genes, we found significant changes in transcription rates in *ptpB* lacking cells for some but not all SarA-regulated genes. While transcription of the *hld* encoding *RNAIII* was affected by the *ptpB* deletion during the exponential growth phase and early stationary phase (Figure 7b), this was neither the case with *hla* nor *splB* (Figure 7a,c). Notably, exponential growth phase cells of the *ptpB* mutant produced lower *RNAIII* transcript rates than wild-type cells, while the opposite was encountered with early stationary phase cells, suggesting PtpB to modulate the transcription of the *agr* locus in SA564 by several means.

One potential factor contributing to this phenomenon could be AgrA, the response regulator of the *agr* locus, and the main transcription factor driving *RNAIII* transcription [35]. Since AgrA is also known to control the expression of the phenol-soluble modulin operons *psmα* and *psmβ* [36], we additionally tested whether *psmα* transcription in SA564 is altered by the *ptpB* deletion (Figure 7d). In line with our *RNAIII* transcription data (Figure 7b), we observed significantly increased *psmα* transcript rates in early stationary phase cells of the Δ*ptpB* mutant, suggesting that PtpB might modulate *RNAIII* and *psmα* transcription in stationary phase cells via AgrA.

To support our transcriptional findings indicating that PtpB does not markedly alter the expression of α-hemolysin in SA564, we tested the hemolytic activity of our strain triplet on sheep blood agar plates (Figure 7e,f). In line with our *hla* transcription findings, we did not encounter clear differences in the hemolytic areas surrounding the growth zones for all three SA564 derivatives.

Together with the observations that SarA was not identified to be phosphorylated at arginine residues [6,7], our transcriptional findings made for the Sar box-containing genes *hla*, *spa*, and *splB* suggest that PtpB is not likely to directly modulate SarA activity, albeit of the fact that the transcription rates of other Sar box containing genes such as *aur*, *hld*, and *nuc* (Figure 2c, Figure 3, and Figure 7b) were markedly affected by the *ptpB* deletion. The differences in transcription rates observed for the latter genes between the wild-type and the *ptpB* deletion mutant might be attributed to other direct PtpB mediated effects on regulatory factors such as MgrA and AgrA, and/or indirect PtpB effects on regulators such as ArlR, CodY, SaeR, SarR, SarV, SarX, and SarZ, which all were found to be expressed to different extents in wild-type and *ptpB* deletion mutant cells of *S. aureus* strain COL under non-stress conditions [6].

## 3. Conclusions

Protein posttranslational modifications such as reversible phosphorylation by kinases/phosphatases is a common mechanism employed by bacteria and eukaryotes to modulate the activity of enzymes and regulatory molecules, which is also utilized by *S. aureus* to adjust central metabolic pathways and virulence factor synthesis [37]. The serine/threonine protein kinase-phosphatase pair Stk1-Stp1 is, for instance, known for its ability to modulate the activities of the SarA family transcription factors MgrA, SarA, and SarZ [38], with Stp1 promoting infectivity, while Stk1 attenuates infectivity [5,38]. Stk1-driven phosphorylation of the catabolite control protein A was additionally shown to inhibit the DNA-binding capacity of the master regulator of carbon catabolite repression in *S. aureus*, thereby modulating the expression of metabolic and virulence genes [39], and infectivity [40]. Our findings, presented here and elsewhere [8], demonstrate that the deletion of *ptpB* in the clinical *S. aureus* isolate SA564 alters the transcription of various genes/operons whose products are involved in stress adaptation and infectivity, suggesting that PtpB-driven removal of phosphates from arginine phosphosites is another posttranscriptional mechanism utilized by this pathogen to fine-tune the expression and activity of its virulon, in order to successfully adapt to the diverse host environmental conditions encountered by the bacterium during infection. Given the clear impact of PtpB on the transcription of specific virulence determinants shown here, its impact on *S. aureus* to cause disease in mice [8], and the fact that several low molecular weight protein tyrosine phosphatase inhibitors are currently in development to combat diseases such as cancer, diabetes/obesity, and bacterial infections [41,42,43,44,45], PtpB might constitute an additional interesting target for drug development against this notorious human nosocomial pathogen.

## 4. Materials and Methods

### 4.1. Bacterial Strains, Media, and Growth Conditions

The bacterial strains used in this study are listed in Table 1. *S. aureus* isolates were plated on Tryptic Soy Agar (TSA; BD, Heidelberg, Germany), or grown in Tryptic Soy Broth (TSB; BD) medium at 37 °C and 225 rpm with a culture to flask volume of 1:10.

### 4.2. Human Whole Blood Phagocytosis Assay

The uptake of *S. aureus* cells by PMNs in whole blood was performed essentially as described in [46]. Overnight cultures of *S. aureus* strains SA564, SA564 Δ*ptpB*, and SA564 ∆*ptpB::ptpB* were inoculated into fresh TSB to an optical density at 600 nm (OD_600_) of 0.05 and grown at 37 °C and 225 rpm to mid-exponential growth-phase (i.e., 2 h). Cultures were centrifuged at 10,000× *g* for 5 min, bacterial pellets washed three times with phosphate buffered saline (PBS; Thermo Fisher, Dreieich, Germany), and subsequently stained with a 50 µM carboxy fluorescein diacetate succinimidyl ester (CFSE; Invitrogen, Darmstadt, Germany)-PBS solution for 15 min at 37 °C and 1000 rpm. CFSE-stained bacterial cells were afterwards washed again three times with PBS to remove unbound dye, and adjusted to an OD_600_ of 1. Fresh human whole blood was withdrawn from healthy donors and anticoagulated with lithium heparin (S-Monovette; Sarstedt, Nümbrecht, Germany). The PMN contents of the blood samples were determined using the RAL DIFF-QUICK kit (RAL Diagnostics, Martillac, France) according to the manufacturer’s recommendations, and blood samples were substituted with fluorescent-labeled bacteria at an MOI of 50 per PMN. Infected blood samples were cultured in the dark at 37 °C and 1000 rpm for 30 min and subsequently placed into 5 mL round bottom polystyrene tubes (BD). Erythrocytes were lysed by adding FACS lysis solution (BD), and lysed cell debris was removed by centrifugation at 450× *g* for 5 min. The cell pellets were resuspended in PBS supplemented with 2% fetal calf serum (PAA, Pasching, Germany) and 0.05% sodium azide, and subjected to flow cytometry using a FACSCalibur (BD) system. PMNs were gated using the CellQuest Pro Software version 4.02 (BD), and the mean fluorescence intensity (MFI) per PMN was recorded, indicating the number of bacteria that were attached to or ingested by the leukocyte.

### 4.3. Extracellular DNase-, Hemolytic- and Proteolytic Activity Assays

Overnight cultures of the *S. aureus* SA564 strain triplet were adjusted for all three assays with fresh TSB to an OD_600_ of 12. For extracellular DNase activity measurements, 5 µL of the adjusted bacterial suspensions were spotted on DNase-Test-Agar plates (Carl Roth, Karlsruhe, Germany) and incubated for 24 h at 37 °C. Lytic zones were visualized by overlaying the agar with 1N HCl to precipitate undigested DNA. The hemolytic activities of the bacterial cell suspensions were tested by spotting 5 µL aliquots of the bacterial suspensions on TSA II plates supplemented with 5% Sheep Blood (BD), and diameters of hemolytic zones were determined after 24 h of incubation at 37 °C. The proteolytic activity of the bacterial cell suspensions was determined by spotting 2 µL aliquots of the bacterial suspensions on TSA plates supplemented with 10% skim milk (BD). Variations in zones of proteolysis were evaluated after incubating the plates for 48 h at 37 °C.

### 4.4. Triton X-100 Induced Autolysis Assay

Triton X-100 induced autolysis of *S. aureus* was assayed using a modified version of the protocol described in [47]. Cells of *S. aureus* SA564 and its *ptpB* deletion mutant were cultured in TSB at 37 °C and 225 rpm to an OD_600_ of 0.7, washed twice in ice-cold water, and resuspended in 0.05 M Tris-HCl (pH 7.2) containing 0.01% (vol/vol) Triton X-100 (Merck, Darmstadt, Germany). The cell suspensions were incubated at 30 °C and 225 rpm and the OD_600_ measured every 30 min. Triton X-100 induced autolysis was determined as the decline of optical density versus time and is expressed as the percent of the initial optical density.

### 4.5. Lysostaphin Induced Autolysis Assay

The lysostaphin-induced autolysis of *S. aureus* was assayed as described in [48]. Cells of *S. aureus* SA564 and SA564 Δ*ptpB* were cultured in TSB at 37 °C and 225 rpm to an OD_600_ of 3, washed twice in PBS, and resuspended in PBS to an OD_600_ of 1. Cell suspensions were supplemented with 250 ng/mL of lysostaphin (Dr. Petry Genmedics GMBH, Reutlingen, Germany) and incubated under static conditions at 30 °C for 60 min. The OD_600_ was measured every 10 min. Lysostaphin-induced autolysis was determined as the decline of optical density versus time and is expressed as the percent of the initial optical density.

### 4.6. qRT-PCR Analyses

RNA isolation, cDNA synthesis, and qRT-PCR were carried out as previously described [49], using the primer pairs listed in Table 2. Transcripts were quantified in reference to the transcription of gyrase B using the 2^−^^ΔCt^ method [50].

### 4.7. Statistical Analyses

The statistical significance of changes between groups was determined using the GraphPad software package Prism 6.01. *p* values < 0.05 were considered statistically significant.

## Figures and Tables

**Table 1:** Strains used in this study.

Strain	Description ^1^	Reference
SA564	*S. aureus* clinical isolate, wild type	[12]
SA564 Δ*ptpB*	SA564 Δ*ptpB::*lox66-*erm*(B)-lox71; Erm^R^	[8]
SA564 Δ*ptpB::ptpB*	*cis*-complemented SA564 Δ*ptpB* derivative	[8]

^1^ Erm^R^, erythromycin-resistant.

**Table 2:** qRT-PCR primer used in this study.

Gene Target	Primer	Sequence (5′-3′)
*atlR*	forward	AACTTATTACACTGACTAACAATG
	reverse	TGTCCAAATCTTCTATTCACTAA
*aur*	forward	AATAGTATTGACGGTGGATTT
	reverse	AATGCTGATAATTTACCTTGATG
*ebh*	forward	GTAATAATGAACAGACTGAGAATC
	reverse	AGCGGATAATGATTGACTATT
*fmtB*	forward	GATGCTTCAAGAATTACAACAA
	reverse	ATCCTGAGAATAGACCTACAT
*gyrB*	forward	GACTGATGCCGATGTGGA
	reverse	AACGGTGGCTGTGCAATA
*hla*	forward	AACCCGGTATATGGCAATCAACT
	reverse	CTGCTGCTTTCATAGAGCCATTT
*hld*	forward	AGGAGTGATTTCAATGGCACAAG
	reverse	TGTGTCGATAATCCATTTTACTAAGTCA
*lrgA*	forward	GCCGGTATCTCAGTTGTTAACTCTT
	reverse	AAATGGTGCTTGGCTAATGACAC
*lytN*	forward	CTATTGTCTTAAATGGTGATTATG
	reverse	ATCTAAACTTTGGAACTTCATTA
*nuc*	forward	TAGCTCAGCAAATGCATCACAA
	reverse	GAACCACTTCTATTTACGCCATTATCT
*psma*	forward	ATCAACAACTCATCACTATGTTAAATCAAC
	reverse	GCCATCGTTTTGTCCTCCTGT
*ptpB*	forward	AGCCCATTAGCGGAAAGTATTG
	reverse	AAATTGATGATTTGGCATAACCTCT
*spa*	forward	TACTTATATCTGGTGGCGTAA
	reverse	GGTCGTCTTTAAGACTTTGA
*splB*	forward	AAGGTAATGGTGGTATTTATTC
	reverse	GAATGACTGATACATCTTCTTTA

## Data Availability

The datasets generated during and/or analyzed during the current study are available from the corresponding author on reasonable request.
